# Machine learning identifies risk factors associated with long-term opioid use in fibromyalgia patients newly initiated on an opioid

**DOI:** 10.1136/rmdopen-2024-004232

**Published:** 2024-05-20

**Authors:** Carlos Raúl Ramírez Medina, Mengyu Feng, Yun-Ting Huang, David A Jenkins, Meghna Jani

**Affiliations:** 1 Centre for Epidemiology Versus Arthritis, Centre for Musculoskeletal Research, The University of Manchester, Manchester, UK; 2 Division of Informatics, Imaging and Data Science, The University of Manchester, Manchester, UK; 3 NIHR Manchester Biomedical Research Centre, Manchester University NHS Foundation Trust, Manchester Academic Health Science Centre, Manchester, UK; 4 Department of Rheumatology, Salford Royal Hospital, Northern Care Alliance, Salford, UK

**Keywords:** fibromyalgia, risk factors, machine learning

## Abstract

**Objectives:**

Fibromyalgia is frequently treated with opioids due to limited therapeutic options. Long-term opioid use is associated with several adverse outcomes. Identifying factors associated with long-term opioid use is the first step in developing targeted interventions. The aim of this study was to evaluate risk factors in fibromyalgia patients newly initiated on opioids using machine learning.

**Methods:**

A retrospective cohort study was conducted using a nationally representative primary care dataset from the UK, from the Clinical Research Practice Datalink. Fibromyalgia patients without prior cancer who were new opioid users were included. Logistic regression, a random forest model and Boruta feature selection were used to identify risk factors related to long-term opioid use. Adjusted ORs (aORs) and feature importance scores were calculated to gauge the strength of these associations.

**Results:**

In this study, 28 552 fibromyalgia patients initiating opioids were identified of which 7369 patients (26%) had long-term opioid use. High initial opioid dose (aOR: 31.96, mean decrease accuracy (MDA) 135), history of self-harm (aOR: 2.01, MDA 44), obesity (aOR: 2.43, MDA 36), high deprivation (aOR: 2.00, MDA 31) and substance use disorder (aOR: 2.08, MDA 25) were the factors most strongly associated with long-term use.

**Conclusions:**

High dose of initial opioid prescription, a history of self-harm, obesity, high deprivation, substance use disorder and age were associated with long-term opioid use. This study underscores the importance of recognising these individual risk factors in fibromyalgia patients to better navigate the complexities of opioid use and facilitate patient-centred care.

**WHAT IS ALREADY KNOWN ON THIS TOPIC**

**WHAT THIS STUDY ADDS**

- Using machine learning methods alongside logistic regression was advantageous in analysing non-linear relationships and reducing sensitivity to multicollinearity in this nationally representative study across 28 552 fibromyalgia patients newly initiated on opioids in the UK.
- This study revealed a strong association between a higher risk of long-term opioid use and prescribing factors such as initial daily morphine milligram equivalent dosage, individual factors such as previous history of self-harm and attempted suicide, high body mass index, substance abuse and sociodemographic factors such as deprivation and age.

**HOW MIGHT THIS STUDY IMPACT RESEARCH, PRACTICE OR POLICY**

- This study highlights prescribing, individual and sociodemographic factors associated with long-term opioid use in fibromyalgia patients.
- In patients with these risk factors, exercising more caution in opioid prescribing, more vigilant monitoring as well as prioritisation of non-pharmacological interventions would enable a more of a personalised approach and improve health outcomes in these individuals.

## Introduction

 Fibromyalgia, a chronic pain disorder, affects between 1.2% and 5.4% of the population.^1^ It is characterised by widespread pain lasting over 3 months in at least four of five body regions. It is often associated with emotional distress and functional disability,^2^ significantly impacting quality of life.^3 4^ Despite its high prevalence worldwide,^5^,^9^ there is no single optimal treatment for fibromyalgia and effective pain-relieving therapies remain limited.^10^ Although recent national^11 12^ and international guidelines,^413^,^15^ including those from the US Centers for Disease Control and Prevention (CDC) and the National Institute for Health and Care Excellence, advise against the prescription of opioids for chronic pain management, opioids continue to be used regularly to alleviate pain in this population. This phenomenon may stem from the restricted availability of alternative analgesic options and the absence of disease-modifying drugs for fibromyalgia, posing a significant challenge for both healthcare professionals and patients.^4 9 16^

Frequency of long-term opioid use is particularly high in fibromyalgia patients even compared with other musculoskeletal conditions. In a recent study with nationally representative data from the UK, we reported that the proportion of patients with fibromyalgia newly initiated on opioids that become long-term users was as high as one in three.^17^ This is of concern as long-term use of opioids has been associated with considerable adverse events, including hospitalisations due to side effects, dependence, overdose, hyperalgesia, immune dysfunction and death.^18^

Published literature and previous cohort studies have reported a variety of risk factors associated with long-term opioid use (also termed persistent use), primarily focusing on postsurgical populations in North American populations.^19^,^22^ While these studies, conducted in a different cultural context and setting, contribute to our overall understanding of postoperative opioid use, the current literature lacks a clear consensus regarding the specific individual, prescribing and contextual risk factors that contribute to long-term opioid use for populations with fibromyalgia, a condition which by itself has been identified as a patient-level risk factor for prolonged use of opioid medications following surgery.^19 23 24^ Additionally, some European studies have aimed to identify factors associated with opioid use in chronic non-cancer pain,^25^ yet these studies have not specifically evaluated patients with fibromyalgia who have differential factors associated with long-term opioid use.

Identifying the specific patient, prescribing, and sociodemographic factors associated with long-term term opioid use within this population is the first step towards developing targeted interventions for deprescribing and imperative for improving future patient outcomes. Thus, the aim of this study was to evaluate the risk factors associated with long-term opioid use in patients with fibromyalgia newly initiated on opioids.

## Methods

### Source of data

A retrospective cohort study was conducted using data from the Clinical Practice Research Datalink (CPRD) GOLD between 1 January 2006 and 31 August 2021. CPRD is a national database of deidentified electronic health records within primary care, representative of the general UK population with regards to age, sex, deprivation and geographical spread.^26^ Records include clinical details, such as medical diagnoses and prescribed medications, in addition to demographic data, information on preventative care and lifestyle choices. Medical history data entered on the General Practice (GP) system, including symptoms, signs and diagnoses, is coded using Read codes and can be used to identify clinical diagnoses.^27^ This data set was linked to English Indices of Multiple Deprivation (IMD) 2019^28^ and to the Hospital Episode Statistics (HES)^29^ database to retrieve ethnicity information.

### Eligibility criteria and study design

This study included adult patients diagnosed with fibromyalgia without prior cancer who were new users of opioids. Patients were considered to have fibromyalgia if their medical records indicated a diagnosis that matched our predefined Read Code list (online supplemental table 1) at any time before the first prescription until 6 months following the new opioid prescription. This extended timeframe was employed to account for a potential delay in the entry of Read Codes following the initial diagnosis. New opioid users were defined as patients who had not been prescribed opioids in at least 2 years preceding the index date, defined as the date of the first opioid prescription. Exclusion criteria included patients with (1) those under the age of 18 at the index date, (2) any history of cancer based on Read Codes within 5 years prior to the index date and (3) those who were prescribed methadone at initiation (since in the UK this is primarily used for treating opioid addiction and not commonly prescribed by general practitioners for pain). Patients were included in this study if their first opioid prescription occurred within 6 months prior to the diagnosis of fibromyalgia or at any time after the diagnosis. Patients were included in the cohort until one of the following events occurred: if they experienced a long-term opioid use event, their departure from the general practice, end of follow-up period or death at which point they were censored.

### Outcome

For this analysis, the most commonly used definition of long-term opioid use in the literature was used, defined as ‘at least three opioid prescriptions issued within a 90-day period from the first new opioid prescription’, or ≥1 opioid prescription lasting at least 90 days, in the first year of follow-up, not including the first 30 days after the index date to allow for the treatment of acute pain.^2430^,^32^

### Covariates

The baseline characteristics of the patients included the age of the patient at opioid initiation, gender, ethnicity and comorbidities. The Charlson Comorbidity Index was used to measure comorbidities based on data from the 5 years prior to the index date. Patient ethnicity data were obtained from CPRD linked data with HES Admitted Patient Care. Based on published literature and clinical knowledge, we also included patient-level characteristics such as history of substance use disorder, depression, attempted suicide or self-harm, alcohol and drug dependence and morphine milligram equivalents (MME) per day of the first opioid prescription on the index date. Daily prescription data were prepared using a drug preparation algorithm published previously.^33^ MME per day was calculated multiplying the daily prescription dose with the corresponding analgesic ratio as outlined by the CDC^24 34^ and was categorised as low: <50 MME/day; medium: 50–119 MME/day; high: 120–199 MME/day and very high: ≥200 MME/day.^24^

The patient’s body mass index (BMI) was calculated based on the closest weight and height measurements to the index date for each individual. BMI was categorised following the classification published by the WHO, which encompasses a range of categories, including underweight (BMI less than 18.5), normal weight (BMI: 18.5 to <25), overweight (BMI: 25 to <30), obese (BMI: 30 to <40) and morbidly obese (BMI of 40 or higher).^35^ IMD, a composite measure that combines information on different domains of material deprivation—income, employment, education and skills, health, housing, crime, access to services and living environment—was also included in the analysis. The IMD, as provided by CPRD, is linked to the practice postcode of the patients at the lower layer super output areas (LSOAs) in England and Wales for 2019 and is divided into five quintiles, with the first quintile being the least deprived quintile and quintile five being the most deprived.^36^ LSOAs are part of the UK’s National Statistics geographical hierarchy, designed to facilitate the reporting and analysis of small area statistics.^37^

Since the impact of daily MME at initiation is not linear, in the logistic regression model, the opioid strength at initiation was divided into a series of discrete intervals accounting for dose, opioid type and sequence of use.^24^ The continuous predictors (eg, age, BMI, mean daily MME) were kept as continuous for the random forest and Boruta models, as these models are more robust to outliers and can learn non-linear relationships between our continuous predictors and long-term opioid use outcome. Implausible and missing values grouped into a separate category for BMI, IMD and ethnicity.

### Statistical analysis

A multivariable logistic regression model was constructed to identify potential risk factors related to long-term opioid use in patients with fibromyalgia using the *tidymodels* workflow in R V.4.2.2. The significance of each predictor variable was assessed using coefficient magnitude and adjusted ORs (aOR), with 95% CIs reported. Given the frequent coexistence of fibromyalgia with various forms of arthritis, such as osteoarthritis (OA), rheumatoid arthritis (RA), systemic lupus erythematosus (SLE), psoriatic arthritis (PsA) and), we assessed the presence of these conditions among the participants in our study. Furthermore, given the previous association of major surgery and long-term opioid use from our previous work in patients with non-cancer pain,^24^ the frequency of this within 1 year of index date was evaluated. Univariable analysis was applied to assess the impact of these covariates on the risk of patients developing long-term opioid use. Additionally, we conducted a sensitivity analysis, excluding patients with other musculoskeletal diseases and major surgery within 1 year prior to index date, to test the robustness of our findings.

A random forest classification algorithm and a Boruta feature selection approach, a wrapper built around the random forest, were also performed to assess the relationship between the covariates and the long-term opioid use outcome. Boruta is a feature selection machine learning algorithm specifically designed for identifying relevant predictors in high-dimensional data sets.^20^ It employs a random forest-based approach to compare the importance of each feature against that of random shadow variables that have no relationship with the outcome or any other predictors in the data set. Random forest and Boruta calculated feature importance scores, which measured how much impact each variable had in predicting long-term opioid use. Items with the highest feature importance scores were the factors with a higher association to the outcome. Compared with logistic regression classification model, random forest variable importance measures covered both the effects of individual predictor variables as well as multivariate interactions with other predictor variables. In the random forest model, the mean decrease accuracy (MDA) index to measure the variables’ importance was used. The *randomForest* (V.4.7–1.1) and Boruta (V.8.0.0) libraries were used in R V.4.2.2. Electronic prescription data were prepared using the framework provided in our previous work.^33^ Data processing and cleaning were conducted in Stata V.13.1 (StataCorp LLC, College Station, Texas). Patients were integral collaborators in the design of the research questions and will continue to be actively engaged throughout the dissemination plans of our research. The study was approved by the CPRD’s Independent Scientific Advisory Committee (approval number: 20_000143).

### Role of the funding source

The study sponsors, including the FOREUM Career Research Grant and the National Institute for Health Research, played no role in the design of the study, the collection, analysis or interpretation of data, the writing of the report or the decision to submit the paper for publication. The research was independently conducted by the authors and the funding sources had no influence on the study’s content of this manuscript.

## Results

### Baseline characteristics

**Figure 1:**
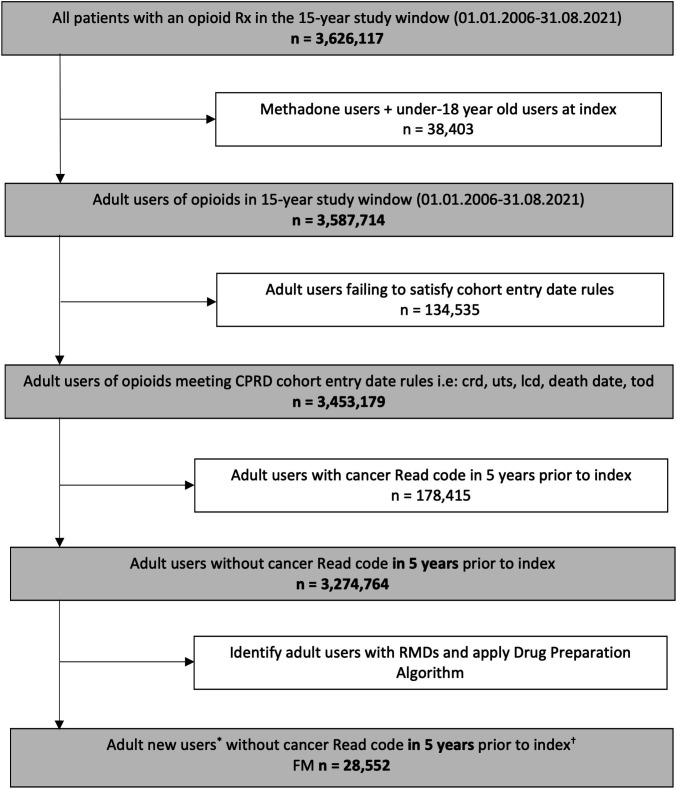
Flowchart for CPRD opioid study cohort creation (15-year study window). *Abbreviations*: *CPRD*, Clinical Practice Research Datalink; *RMDs*, Rheumatic and musculoskeletal diseases; *FI*, Fibromyalgia; *crd*, date the patient’s current period of registration with the practice began; *lcd*, date of the last collection of data for the practice; tod, date the patient transferred out of the practice; uts, up to standard/ date at which the practice data was deemed to be of research quality. ^†^ The number of patients referred to unique patient IDs. A patient could be a new opioid user again when the time window between opioid prescriptions was more than two years. In this study, only the first prescription for each individual was included. ^*^ The follow-up of eligible individuals started with the current registration date that the practice also met data quality metric (i.e., up to standard date) and ended up with the earliest time of transfer out dates, death dates, and last collection dates. For those who took opioids, the start of follow-up also accounted for the time when an individual had an opioid prescription up to 6 months before or any time after an RMD diagnosis.

The study included 28 552 adult new opioid patients with fibromyalgia (figure 1). Within the first year, 26% (7369 patients) transitioned to long-term opioid use, using the definition above. Of fibromyalgia patients with new opioid use, 84.8% were women and 15.2% were men. This distribution is consistent with existing research on the demographics of fibromyalgia.^38^,^40^ Among patients with available ethnicity data in HES, 94% were of white ethnicity. The largest age group comprised those aged 45–54 years (28.0%), followed by those aged 55–64 years (22.0%). At the initiation of opioid use, the median daily dose of MME was 21.36 (IQR 9.60–27.55).

Among patients who developed long-term opioid use, 26.7% were classified as obese (BMI: 30 to <40), representing the largest proportion, followed by 17.9% who were categorised as overweight (BMI: 25 to <30). In contrast, for patients who did not develop long-term opioid use, the predominant category was overweight (BMI: 25 to <30), encompassing 23.8% of the cohort. Of the patients who developed long-term opioid use, 6.0% had a history of self-harm and attempted suicide, compared with 2.7% in the group that did not develop long-term opioid use. The prevalence of depression in patients with fibromyalgia was high in those who went onto develop long-term opioid use and those who did not) (26.4% vs 27% respectively).

In patients with fibromyalgia who did not develop long-term opioid use and where data were available, the largest category belonged to the least deprived socioeconomic groups (quintile 1, 21%; quintile 2, 22.2%). In contrast, patients who did transition to long-term opioid use were predominantly from the most deprived socioeconomic group (Quintile 5, 25%).

Codeine was the most commonly initiated opioid in our cohort (18 379, 64.3%). Tramadol followed as the second most common, representing 13.8% among non-long-term users and increasing to 24.3% among long-term users. While morphine use was relatively low in non-long-term cases (1.1%), its use was higher in 6.2% among long-term users. Higher percentages of buprenorphine (3.5%), oxycodone (2.1%) and fentanyl (1.7%) were also found among long-term users compared with non-long-term users (table 1).

**Table 1:** Baseline characteristics of new users of opioids (n=28 552) grouped by long-term opioid use

Characteristic	No long-term opioid use		Long-term opioid use	
	(Total patients n=21 183)		(Total patients n=7369)	
Female, n (%)	18 005	84.90%	6203	84.20%
Male, n (%)	3178	15.10%	1166	15.80%
Age (years), mean (SD)	49.38	14.2	49.79	13.2
Age group (years) at index, n (%)				
18–24	890	4.20%	193	2.60%
25–34	2529	11.90%	754	10.20%
35–44	4280	20.20%	1616	21.90%
45–54	5760	27.20%	2223	30.20%
55–64	4678	22.10%	1600	21.70%
65–74	2199	10.40%	718	9.70%
75–84	712	3.40%	215	2.90%
≥85	135	0.60%	50	0.70%
Ethnicity, n (%)				
White	6676	93.60%	2014	95.80%
Asian	261	3.70%	45	2.10%
Black	82	1.10%	14	0.70%
Other	81	1.10%	18	0.90%
Mixed	36	0.50%	11	0.50%
BMI, n (%)				
Underweight (<18.5 kg/m^2^)	295	1.90%	74	1.50%
Normal weight	4671	29.60%	1117	22.10%
Overweight	5049	32.00%	1321	26.20%
Obese	4838	30.70%	1969	39.00%
Morbidly obese (≥40 kg/m^2^)	929	5.90%	570	11.30%
Mental health and behavioural risk factors, n (%)				
History of suicide and self-harm, n (%)	581	2.70%	441	6.00%
History of depression, n (%)	5545	26.20%	1987	27.00%
History of alcohol dependence, n (%)	225	1.10%	139	1.90%
History of substance use disorder, n (%)	156	0.70%	144	2.00%
Charlson Comorbidity Index, n (%)				
Low score (0)	16 067	75.80%	5578	75.70%
Medium score (1–3)	5021	23.70%	1746	23.70%
High score (≥4)	95	0.40%	45	0.60%
Index of multiple deprivation, n (%)				
1 (least deprived)	1530	20.60%	325	15.00%
2	1656	22.20%	381	17.50%
3	1618	21.70%	495	22.80%
4	1426	19.20%	433	19.90%
5	1214	16.30%	537	24.70%
Mean daily MME, n (%)				
Low (<50)	21 000	99.10%	6770	91.90%
Medium (50–119)	168	0.80%	446	6.10%
High (120–199)	<10	0.00%	106	1.40%
Very high (≥200)	<10	0.00%	47	0.60%
Drug substance, n (%)				
Codeine	14 790	69.80%	3580	48.60%
Tramadol	2929	13.80%	1792	24.30%
Dihydrocodeine	2836	13.40%	922	12.50%
Morphine	226	1.10%	456	6.20%
Buprenorphine	200	0.90%	260	3.50%
Oxycodone	91	0.40%	154	2.10%
Fentanyl	28	0.10%	125	1.70%
Dextropropoxyphene/paracetamol	41	0.20%	21	0.30%
Tapentadol	10	0.10%	42	0.60%
Others	32	0.30%	17	0.10%
Missing, n (%)				
Missing IMD	13 739	64.90%	5198	70.50%
Missing BMI	5401	25.50%	2318	31.50%
Missing ethnicity	14 041	66.30%	5273	71.60%

Proportions are presented as percentage of non-missing data.

Some patients had missing data for certain variables, which is reported at the end of the table.

Other drug substances include: meptazinol, pethidine, dipipanone/cyclizine, hydromorphone and pentazocine.

Values<10 patients are suppressed to comply with Statistical Disclosure Control.

Opioid dosage: low MME defined as<50 MME/day,. Mmedium MME (50–119 MME/day), Hhigh MME (120–199 MME/day), very high MME (≥200 MME/day).

BMIbody mass indexIMDIndex of Multiple DeprivationMMEmorphine-milligram equivalent

### Logistic regression

**Figure 2:**
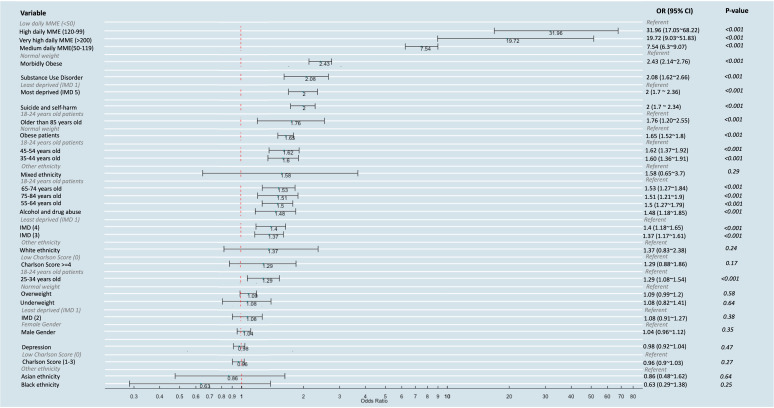
Factors associated with long-term opioid use in fibromyalgia patients ranked by adjusted Odds Ratio. IMD, Index of Multiple Deprivation; MME, morphine milligram equivalents.

Patients with a high initial daily MME dose (120–199 MME/day) exhibited the highest aOR of 31.96 (95% CI 17.05 to 68.21, p<0.001) when compared with those with a low mean daily MME (<50 MME/day). Morbidly obese (BMI of 40 or higher) individuals faced a 2.43-fold higher likelihood of long-term opioid use compared with those with a normal BMI (aOR: 2.43, 95% CI 2.14 to 2.76, p<0.001). Additionally, patients with a history of substance use disorder were about two times as likely to develop long-term opioid use (aOR: 2.08, 95% CI 1.62 to 2.66, p<0.001) and those identified as most deprived (Index of Multiple Deprivation (IMD quintile 5) were associated with a doubling of risk of long-term opioid use compared with those least deprived (aOR: 2.01, 95% CI 1.70 to 2.36, p<0.001). Individuals with a history of suicide and self-harm were also two times as likely to develop long-term opioid use (aOR: 2.0, 95% CI 1.70 to 2.34, p<0.001). Finally, patients aged 85 years or older had a 1.76 times higher risk of long-term opioid use (aOR:1.76, 95% CI 1.20 to 2.55, p<0.001) compared with the youngest group (figure 2).

We found 40% of fibromyalgia patients had a concurrent diagnosis of OA, while smaller proportions had diagnoses of RA (1.9%), PsA (0.9%), SLE (0.9%) and AS (0.4%). Approximately 1.1% of our cohort had undergone major surgery during this period. We found no statistically significant results in the univariable analysis assessing the association of OA, SLE and major surgery with long-term opioid use in this population (online supplemental table 2). Sensitivity analysis excluding patients with other musculoskeletal diseases and with major surgery within 1 year prior to index date corroborated the importance of the top variables identified in the previous analysis (online supplemental figure 1).

### Random forest

**Figure 3:**
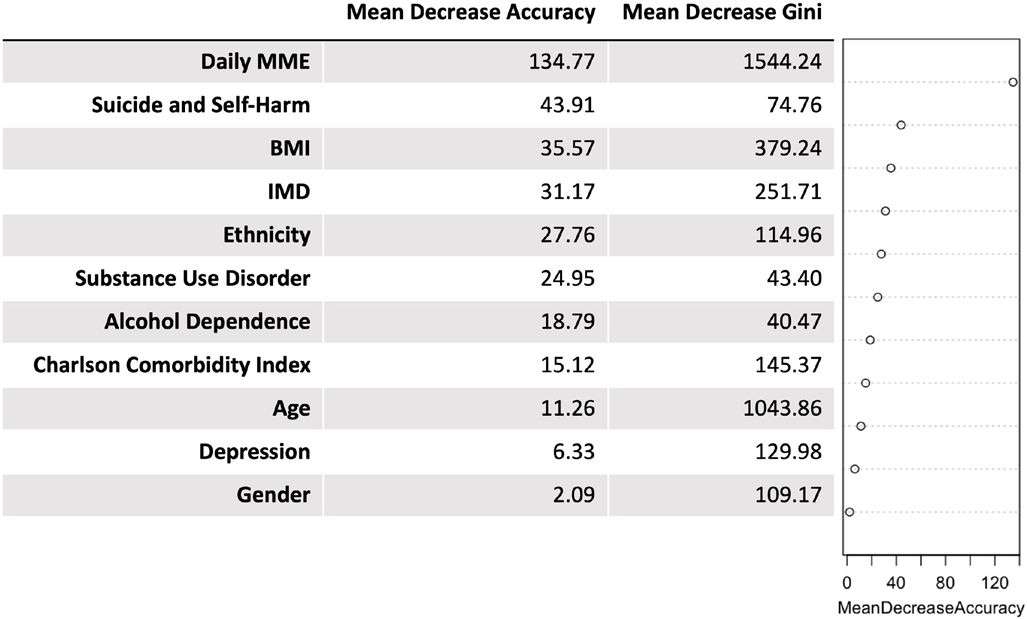
Results from the Random Forest modeland plot ranked by MDA. The most important factor was daily morphine milligram equivalent (MME). *Abbreviations*: BMI, body mass index; IMD, Index of Multiple Deprivation; MDA, mean decrease accuracy.

Using the random forest model, the most important variables ranked by MDA are presented in figure 3. Daily MME dosage stood out as the most important variable, with and MDA of 134.77, followed by history of self-harm and attempted suicide (MDA 43.91), BMI (MDA 35.57) and IMD (MDA 31.17). Ethnicity (MDA 27.76), substance use disorder (MDA 24.95), alcohol dependency (MDA 18.79), Charlson comorbidity score (MDA 15.12) and age (MDA 11.26) also exhibited considerable importance. The analysis placed depression and gender as less influential factors, with MDA scores of 6 and 2, respectively.

### Boruta feature selection

**Figure 4:**
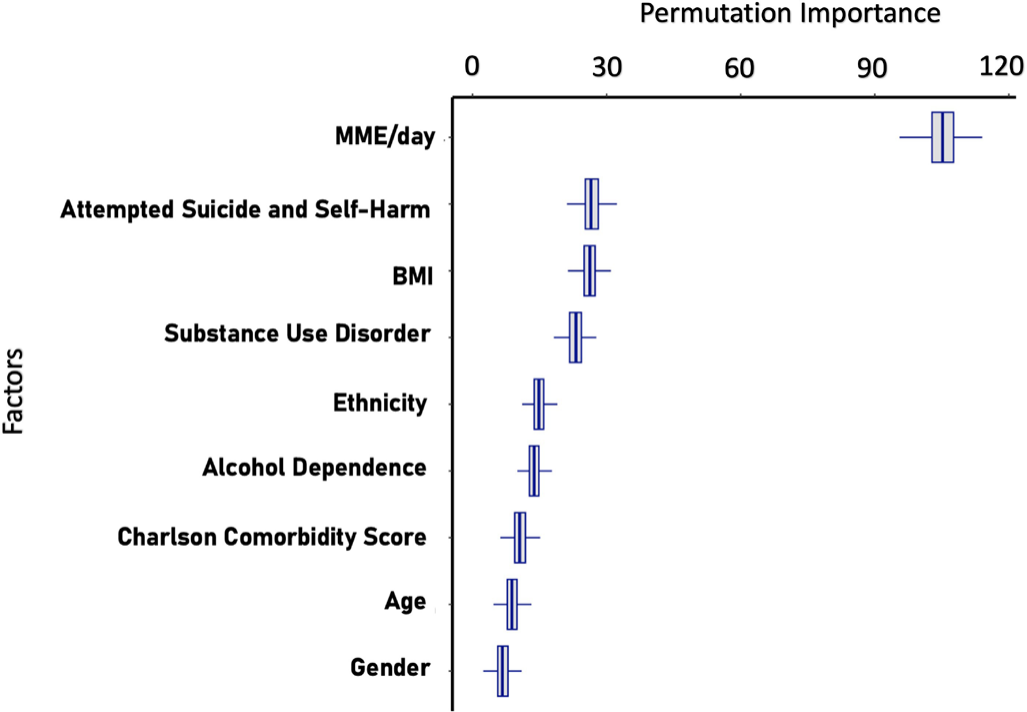
Results from Boruta feature selection model and plot ranked by permutation importance. BMI, body mass index; MME, morphine milligram equivalents.

Boruta Feature Selection algorithm (figure 4) demonstrated a strong association of daily MME at initiation with long-term opioid use in our population with a mean importance (MI) score of 105.34). Additionally, it also highlighted the importance of a history of suicide and self-harm (MI: 26.71), BMI (MI: 26.32) and socioeconomic status as represented by the IMD (MI: 23.13) in their association with long-term opioid use. Variables such as substance use disorder (MI: 14.89), ethnicity (MI: 13.84), alcohol dependence (MI: 10.61), Charlson comorbidity score (MI: 8.91) and age (MI: 6.76), even though still confirmed as important, exhibited lower MI scores. History of depression and gender was not considered to be important features associated with long-term opioid use.

## Discussion

In a nationally representative cohort of fibromyalgia, patients newly prescribed opioids a strong association between the daily MME at initiation and an increased risk of long-term opioid use was found. Additionally, history of attempted suicide and self-harm, obesity and high social deprivation were also ranked as some of the most important factors associated with long-term opioid use by machine learning algorithms, capable of capturing non-linear relationships and interactive patterns in data. To our knowledge, this study represents the first comprehensive analysis of risk factors associated with long-term opioid use among fibromyalgia patients, harnessing a nationally representative dataset.

The consistent top ranking of high initial MME/day dosage across the main analytical methods holds particular importance due to the inclination of higher opioid dosages to foster tolerance, dependence and addiction,^22 23^ alongside a heightened susceptibility to adverse events such as respiratory depression, constipation and nausea. This amalgamation of potential adverse events is compounded by the fact that patients who begin opioid treatment with elevated doses often grapple with multiple health conditions and coprescriptions, which could make this population especially susceptible to harms.^41^

While the higher importance of history of self-harm and attempted suicide in the machine learning algorithms compared with other variables such as patient’s age, comorbidities, substance use disorder and alcohol dependence may initially seem surprising, it aligns with the understanding that mental health factors play a critical role in opioid use patterns for patients with fibromyalgia and vice versa. Individuals with a history of self-harm and suicide may be more vulnerable to long-term opioid use as they seek relief from emotional distress through opioids.^42^ At the same time, a heightened risk of suicide is strongly associated with the consumption of higher doses of prescribed opioids in pain management.^43^ Notably, chronic widespread pain, the cardinal symptom of fibromyalgia, is recognised as a potential contributor to the risk of suicidal thoughts or behaviours by clinical guidelines. The WHO also identifies chronic pain as a patient-level key risk factor for suicide.^44^ Although the results of the analyses cannot establish causation, they do indicate a strong association between a history of suicide and self-harm and prolonged opioid use. The close ties and complex relationships among pain, opioid prescriptions and suicide risk have been documented in the literature^45^,^47^ and underscored the imperative of acknowledging this patient-level factor in opioid management.

Additionally, high BMI and high deprivation were also found to be highly associated with the development of long-term opioid use. Obesity is particularly relevant as it may contribute to the severity of fibromyalgia, increasing fatigue and worsening symptoms.^48^ Furthermore, obesity is suggested to affect how the body metabolises medications^49^ and impacts pain perception. Previous studies have noted a high prevalence of obesity among individuals with fibromyalgia.^48^ Among patients for whom data were available, 40% were classified as either obese or morbidly obese, with an additional 31% falling into the overweight category. Obesity may exacerbate pain in individuals with fibromyalgia by placing added pressure on joints and tissues.^50^ In contrast to individuals with fibromyalgia who have a normal body weight, those who are overweight or obese and have fibromyalgia tend to experience more intense pain, diminished physical abilities, and a lower overall quality of life.^51^,^53^ Obesity has been found to be strongly associated with incident long-term prescription use in previous studies.^54^ Moreover, this factor is also closely linked with social deprivation. Individuals living in economically deprived areas may have limited access to healthier lifestyle options, potentially contributing to higher BMIs.^55^ Socioeconomic status and pre-existing mental health disorders have also been reported to be important indicators of long-term opioid use in the postsurgical population,^19^ though not in patients with fibromyalgia previously.

In this study, depression was not found to be specifically associated to long-term opioid use in the fibromyalgia population, despite being reported as an important factor, along with psychiatric comorbidities, in the risk of opioid use among general patient populations in the USA^56^ and in the UK.^24^ Although a high prevalence of depression in patients with fibromyalgia was found in this study (26.4%), no significant difference was observed between those who developed long-term opioid use and those who did not. The findings of this study suggest that in the complex, multifactorial nature of pain, other variables, such as the ones mentioned earlier, may exert a more influential role in the relationship with long-term opioid usage.

The CDC identified a history of alcohol dependence and substance use disorder as factors that increase the risk of long-term opioid use^57^ in patients, factors that were also confirmed as important but ranked lower in the present study. Patients with a history of substance abuse, obesity, and the use of psychiatric medications are known to have an increased risk of chronic opioid use among those with musculoskeletal conditions like rheumatoid arthritis.^58^ This study implies that a similar relationship may hold true for patients with fibromyalgia. Moreover, opioid use has been reported to increase with age in general adult populations in the USA,^59^ which is in line with the results of our analyses.

Certain limitations warrant acknowledgement. CPRD uses Read Codes to identify different clinical diagnoses, which is the standard method for characterising conditions in UK primary care electronic health records (EHRs).^27^ The present study did not use classification criteria typically employed in prospective observational studies, as this is not done in routine clinical practice. As the cultural context of opioid prescribing varies between countries, caution is advised when extrapolating the findings of this study to other settings.

Additionally, CPRD and routinely collected EHRs do not provide information on the severity of pain at the time of opioid prescription or the specific reasons for prescribing particular opioid doses to individual patients.

In general practice, the decision to prescribe opioids depends on various factors, including the severity of the pain, the underlying cause of the pain, the patient’s medical history and their response to other non-opioid treatments. However, the present analysis was limited to baseline variables available within routinely collected primary care data. Notably, the incorporation of other variables, such as education, unemployment status, severity of mental illnesses, which are often observed in opioid-using fibromyalgia patients,^4 19^ was not possible due to their absence in routinely collected data in general practice.

Furthermore, it is important to acknowledge another limitation inherent in our study, namely, some missing values in both BMI and ethnicity data among the patients included in our analysis, as observed within the CPRD data set. Missing data are a pervasive challenge across EHRs in general, and this limitation underscores the need for continued efforts to enhance data completeness and accuracy within such healthcare databases. It is also important also to highlight that the results reported do not establish causation. The statistical methods employed in this study, such as logistic regression and machine learning algorithms, can identify associations based on patterns in the data, but they cannot prove that one factor causes another. For instance, the prescription of a high MME/ day at initiation could reflect the healthcare provider’s perception of the patient’s pain intensity during initiation.

Nonetheless, the findings of this study hold important implications for the effective management of chronic pain in patients with fibromyalgia. Primarily, these findings highlight an important association between long-term opioid usage and factors such as high MME/day at initiation, history of self-harm, obesity and high levels of deprivation. Finally, the results of the present study could help in identifying specific patient groups that require increased attention and support, particularly in promoting non-pharmacological alternatives where accessible. Clinicians should carefully consider the risks and benefits of prescribing opioids to patients with fibromyalgia that fall within these subgroups before doing so.

The association of BMI and socioeconomic factors with long-term opioid use above other factors underscores the importance of addressing social determinants of health and promoting healthier lifestyles as part of pain management strategies. Additionally, it is important to be aware of the high risk of long-term opioid use in patients with fibromyalgia: under the long-term opioid definition used in this nationally representative study, one in four patients with fibromyalgia became long-term opioid users.

By closely monitoring these subgroups of patients, healthcare professionals can better identify early signs of escalating opioid use, promptly address emerging issues and tailor interventions to reduce potential risks. In healthcare settings where resources are limited, these results could assist healthcare providers in identifying fibromyalgia patients who may benefit from more frequent monitoring. Additionally, they can provide these patients with alternative pain management options, including non-pharmacological interventions, thereby reducing the likelihood of long-term opioid harms and poorer outcomes.

## supplementary material

10.1136/rmdopen-2024-004232 online supplemental file 1

## Data Availability

Data may be obtained from a third party and are not publicly available.
