# Supplementary material for: Machine learning identifies risk factors associated with long-term opioid use in fibromyalgia patients newly initiated on an opioid
Source: RMD Open. 2024 May 20;10(2):e004232. doi: 10.1136/rmdopen-2024-004232 (PMC11308899; doi:10.1136/rmdopen-2024-004232)
Supplement: online supplemental file 1 [file rmdopen-10-2-s001.pdf]

Supplementary Table 1: Read Codes Used for Identifying Fibromyalgia Diagnoses in CPRD GOLD

READCODE	READ TERM
N248.00	Fibromyalgia
N239.00	Fibromyalgia
Ryu7000	[X] Other chronic pain
66n..00	Chronic pain review
1M52.00	Chronic pain
F286.0	Chronic fatigue syndrome
F286000	Mild chronic fatigue syndrome
F286100	Moderate chronic fatigue syndrome
8HkW.00	Referral to chronic fatigue syndrome specialist team
F286.11	CFS - Chronic fatigue syndrome
F286.00	Chronic fatigue syndrome
8HlL.00	Referral for chronic fatigue syndrome activity management
F286200	Severe chronic fatigue syndrome
8Q1..00	Activity management for chronic fatigue syndrome
F03y.12	Myalgic encephalomyelitis
F286.15	Myalgic encephalomyelitis

Supplementary Table 2: Baseline characteristics of painful comorbidities in our study cohort and results from logistic regression analysis.

Characteristic	Total	No long-term opioid use	Long-term opioid use	Adjusted Odds Ratio	P-value
OA	11,297 (39.9%)	8,458 (30%)	2839 (9.9%)	0.94	0.03
RA	547 (1.9%)	368 (1.3%)	179 (0.6%)	1.40	<0.001
PSA	250 (0.9%)	157 (0.6%)	93 (0.3%)	1.68	0.002
SLE	249 (0.9%)	172 (0.6%)	77 (0.3%)	1.27	0.04
AS	101 (0.4%)	57 (0.2%)	44 (0.2)	2.21	<0.001
Major Surgery (one-year prior)	320 (1.1%)	240 (0.8%)	80 (0.3%)	0.95	0.67

Supplementary Figure 1: Sensitivity analysis excluding patients with other musculoskeletal diseases and major surgery within one year prior to the index date. Forest Plot with Top Ranked Variables

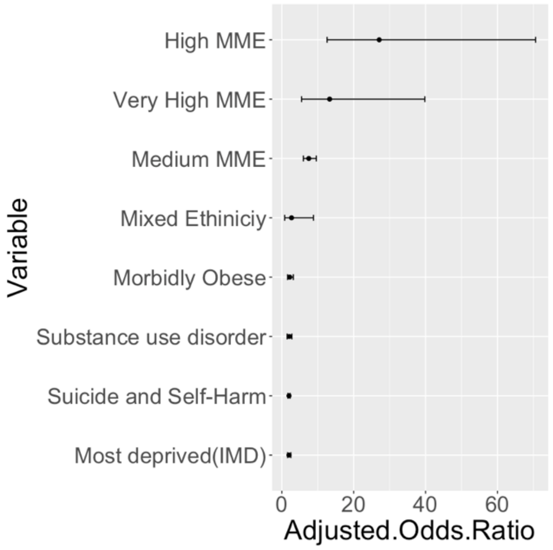

Supplementary Table 3: Sensitivity analysis – Results from multivariate logistic regression excluding patients with other musculoskeletal diseases and major surgery within one year prior to the index date. Table with Odds Ratios and 95% Confidence intervals.

	Adjusted Odds Ratio	95% CI Lower	95% CI Upper
High MME (120-199)	27.1	12.6	70.6
Very High MME (>200)	13.3	5.5	39.8
Medium MME (50-119)	7.5	6	9.6
Mixed Ethnicity	2.7	0.8	8.8
Morbidly Obese	2.2	1.6	3.2
Substance Use Disorder	2.1	1.5	2.8
Suicide and Self-Harm	2	1.7	2.3
Most Deprived (IMD 5)	2	1.6	2.5
White	1.9	0.9	4.5
>= 85 years old	1.9	1	3.5
Obese	1.6	1.1	2.3
45-54 years old	1.6	1.3	2
75-84 years old	1.6	1.1	2.2
IMD 4	1.5	1.2	1.8
35-44	1.5	1.2	1.8
55-64	1.5	1.2	1.8
65-74	1.5	1.2	1.9
IMD 3	1.4	1.1	1.8
Alcohol and Drug Dependence	1.4	1.1	1.9
25-34	1.3	1	1.6
IMD 2	1.1	0.8	1.3
Asian	1.1	0.5	2.9
Normal BMI	1	0.7	1.4
Overweight	1	0.7	1.5
Charlson High Score (>=4)	1	0.5	2
Charlson Medium Score (1-3)	1	0.9	1.1
Depression	1	0.9	1.1
Female	1	0.9	1.1

* The variables presented in this table are ordered by magnitude of Odds Ratios.
